# 

**Published:** 1902-05-31

**Authors:** 


### NOTICE TO CORRESPONDENTS.

All MSS., Letters, Books for Review, and other matters intended for the Editor should be addressed The Editor, "The Hospital," The Hospital Building, 28 & 29 Southampton Street, Strand, W.C.

The Editor cannot undertake to return rejected MS., even when accompanied by stamped directed envelope.

**Notice to Advertisers and Subscribers.**

All Advertisements, Orders for Copies of The Hospital, and Business Communications should be addressed to **THE MANAGER, (Not to the Editor)**, The Hospital, 28 & 29 Southampton Street, Strand, London, W.C.

The Cost of Subscription, post free, is as follows.—Per week, 2½d.; per quarter, 2s. 8d.; per half-year, 5s. 3d.; per annum, 10s. 6d. Foreign:—Per annum, 15s. 2d., payable, in all cases, in advance.

**NOTICE.—Vol. XXXI. of "The Hospital" (October 1901, to March 1902), in handsome cloth binding is now on sale at the office of this Journal, price, 7s. 6d. Cases for binding the Numbers contained in this Volume 1s. 6d. Index to Vol. XXXI., 2½d., post free.**

**The Monthly Part for June will be ready on June 2nd. Price 6d., by post 8d.**

### BOOKS RECEIVED.

CASSELL AND CO.

"Clinical Methods: a Guide to the Practical Study of Medicine." By Robert Hutchison, M.D., M.R.C.P., and Harry Rainy, M.A., F.R.C.P.Ed., F.R.S.E.

W. MATE AND SONS, BOURNEMOUTH.

"Annual Reports of the Medical Officers of Health for Bournemouth and Winton."

WILLIAM BLACKWOOD AND SONS.

"The Way of Escape." A Novel. By Graham Travers (Margaret Todd, M.D.).

GEORGE NEWNES.

"Doctor Therne: Anti-Vaccinist." By H. Rider Haggard.

LONGMANS, GREEN, AND CO.

"Lectures on the Use of Massage and Early Passive Movements in Recent Fractures, etc." By Sir William H. Bennett, K.C.V.O., F.R.C.S.

"The Essentials of Histology." By E. A. Schäfer, LL.D., F.R.S.

ARMY AND NAVY GAZETTE.

"Mentioned in Despatches."

ISBISTER AND CO.

"A Hero of Donegal, Dr. William Smyth." By Frederick Douglas How.

HENRY J. GLAISHER.

"Clinical Lectures on Neurasthenia." By Thomas D. Savill, M.D.

### SCRAPS AND GLEANINGS.

SUNDAY, November 9th, has been fixed as Hospital Sunday for Bury, and Saturday, July 26th, is to be the Hospital Saturday.

THE total cost of the buildings of the new infirmary at Southampton, including the purchase of land, decorating and furnishing, is £89,000. The total number of beds provided for is 300.

THE new convalescent home, which the Birmingham Hospital Saturday Fund are converting to their use out of an old dwelling-house, in place of their present home near Llandudno, is approaching completion. The building will eventually provide accommodation for 250 beds.

### THE STUDENT OF MEDICINE.

### APPOINTMENTS

**W. D. Adams**, M.B., M.S.Edin, appointed District Medical Officer for the parishes of Banham, Eccles, Kenninghall, Quidenham, and Wilby, and also Vaccinator for these parishes. **G. Granville Bothwell**, M.B., C.M.Aberd., appointed Resident Surgeon to the Bonsor Gold Mining Company, Selukwe, Rhodesia, South Africa. **W. H. Eden Brand**, L.R.C.S. and L.R.C.P.Edin., appointed House Surgeon to the Northern Infirmary, Inverness. **Alex. Brownlee**, L R.C.P. and S Edin., L.D.S.Edin., appointed Assistant House Physician to the Infirmary, Cardiff. **W. H. Clayton-Greene**, B.A., M.B., B.C.Cantab., F.R.C.S.Eng., appointed Surgical Registrar to St. Mary's Hospital. **John Henry Harris**, M.D.Durh., M.R.C.S., L S.A., D.P.H.Cantab., appointed Medical Officer of Health for the Dartmouth Port Sanitary Authority. **P. Heffernan**, L.R.C P., L.R.C.S.Edin., L.F.P.S.G., appointed Assistant Medical Officer to the Clonmel Asylum. **William Andrew Hewitson**, M.R.C.S.Eng., L.R.C.P.Edin. appointed Medical Officer to the Workhouse, District Medical Officer, and Public Vaccinator by the Easington Board of Guardians, also Medical Officer to the Infectious Diseases Hospital, Thorpe, near Easington. **Wilfrid Howard**, L.R.C.P.Edin., M.R.C.S., appointed District Medical Officer for the parishes of New and Old Buckenham, and Public Vacinator for the same parishes. **Edwin Hyde**, M.R.C.S, L.R.C.P.Lond., appointed Assistant House Surgeon to the Infirmary, Cardiff. **W. Ernest Thomson**, M.A., M.D., appointed a Surgeon to the Glasgow Eye Infirmary. **William Thyne**, M.A., M.D.Edin., appointed Medical Superintendent to the Isolation Hospital for the Barnet Urban District. **E. S. Worrall**, M.R.C.S., L.R.C.P.Lond., appointed in charge of the Radiographic Department, University College Hospital, London.

**"The Hospital" Institutional Library.**

"Hospitals and Asylums of the World." 4 Vols., with a Portfolio of Plans, complete £12 12s.

"Burdett's Hospitals and Charities, 1902." 5s.

"Cottage Hospitals, General, Fever, and Convalescent." 10s. 6d.

"Hospital Expenditure: The Commissariat." 2s. 6d.

"A Legal Handbook for the Use of Hospital Authorities." 2s. 6d.

"The Cottage Hospital Case Book or Register of Patients." 10s. and 12s 6d.

"The Furnishing and Appliances of a Cottage Hospital." 6d.

All these are published by the SCIENTIFIC PRESS, Ltd., and may be obtained through any bookseller, or direct from the publishers, 28 and 29 Southampton Street, Strand, London, W.C.

Birmingham.

Crown 8vo. paper cover, 6d.

**On Preparation for Operation in Private Houses.**

By STANMORE BISHOP, F.R.C.S.

THE SCIENTIFIC PRESS, LTD., 28 & 29 Southampton Street, Strand, London, W.C.

